# Parenting Experiences in the Context of Parental Bipolar Disorder: A Systematic Review and Meta-Synthesis of the Qualitative Literature

**DOI:** 10.1007/s10567-025-00513-x

**Published:** 2025-02-13

**Authors:** En-Nien Tu, Kate E. A. Saunders, Helen Manley, Fiona Lobban, Steven Jones, Cathy Creswell

**Affiliations:** 1 Department of Psychiatry and Experimental Psychology, University of Oxford, Oxford, UK; 2 Chang Gung Memorial Hospital, Keelung, Taiwan; 3 Chang Gung University, Taoyuan, Taiwan; 4 Department of Psychiatry, University of Oxford, Oxford, UK; 5 Oxford Health NHS Foundation Trust, Warneford Hospital, Oxford, UK; 6 Spectrum Centre for Mental Health Research, Division of Health Research, Faculty of Health and Medicine, Lancaster University, Lancaster, UK; 7 Lancashire and South Cumbria, NHS Foundation Trust, Lancaster, UK; 8 Department of Experimental Psychology, University of Oxford, Radcliffe Observatory, Anna Watts Building, Woodstock Rd, Oxford, OX2 6GG UK

**Keywords:** Family, Relationship, Interaction, Child, Depression, Mania

## Abstract

Parental bipolar disorder (BD) is associated with an increased risk of mental health problems in children. Despite the urgent need for clear guidance on how best to support parents with BD, current research lacks a unified analysis of the challenges and needs faced by these parents and their children. This review aims to explore the impact of BD on experiences of parent–child interactions or relationships to inform effective policies and interventions. Following a preregistered PROSPERO protocol, we searched Medline, Embase, PsycINFO, SCOPUS, and CINAHL for qualitative studies on parents with BD and their children (under 19 years) published since 1994. Each study was independently screened and jointly assessed for quality using the Critical Appraisal Skills Program. Our thematic synthesis entailed coding in NVivo, followed by collaborative theme generation on the Miro platform. We reviewed 19 studies, of which 9 studies reported on parents, 8 on children, and 2 on both parents and children. Our analysis generated four themes: (1) "The multifaceted landscape of parenting with BD," which outlines how mood swings affect parenting in diverse ways; (2) "The evolving dynamic of child-parent relationship amidst parental BD," including how children adapt and grow in understanding and responsibility in response to their parent’s BD; (3) "The dual nature of childcare for parents with BD," which explores how childcare impacts parents’ emotions and motivations, bringing both uplifting and challenging effects; (4) "Navigating parental challenges in the context of BD," highlighting the importance of open communication, self-reflection, and timely, unbiased support to mitigate challenges associated with parental BD. This qualitative synthesis focuses specifically on the parenting experiences of families affected by parental BD. It highlights the complex, dynamic impact of BD on parenting behaviors and children’s coping mechanisms, calling for tailored therapeutic interventions that benefit both parents and children. The scope of our study is limited by factors such as a predominance of Western perspectives and an underrepresentation of fathers’ experiences, highlighting the need for more diverse research in this area.

## Introduction

Bipolar disorder (BD) is a chronic mental health condition characterized by alternating periods of depression and heightened states of mania or hypomania. The population prevalence is estimated to be 1% ((Merikangas et al., 2011). Typically, symptoms begin to emerge in late adolescence or early adulthood and can interfere with significant life milestones, such as forming a family and raising children (Bolton et al., 2021; Grande et al., 2016). Research on how parental BD impacts families is evolving, and there is growing evidence of certain patterns and commonalities. For example, it has been reported that families with a parent with BD often experience less cohesion and more conflict than other families (Shalev et al., 2019; Stapp et al., 2019, 2020). Additionally, children of parents with BD face a significantly higher risk for psychiatric conditions, with a 2- to threefold increased risk for any psychiatric diagnosis and an 11-fold increased risk for developing BD before age 18 compared to children of parents without BD (Lageborn et al., 2024). These risks are particularly elevated for mood disorders when children are exposed to additional vulnerabilities such as parental neglect and emotional mistreatment (Doucette et al., 2016; Koenders et al., 2020). Impairments in functioning can emerge as early as preschool age, with most studies on offspring of parents with BD up to age 19 reporting global and social impairments, though a few show no significant differences (Helmink et al., 2023).

Quantitative studies have been crucial in identifying broad patterns, prevalence, risk factors, and outcomes for families affected by BD. However, they often lack the depth to capture the nuanced and intricate lived experiences of these families. Although some qualitative studies exist, they typically focus on subgroups within the bipolar community, such as hospitalized parents or those with peripartum onset (Anke et al., 2019; Liu et al., 2022; Mulvey et al., 2022; Reupert et al., 2012). Furthermore, the existing qualitative meta-syntheses that have considered the parenting experiences of parents with BD have grouped them with parents dealing with other mental health conditions, such as psychosis and major depressive disorders (MDD) (Dolman et al., 2013; Harries et al., 2023). While these studies identify common challenges such as balancing self-care with childcare, stigma, guilt, and concerns about impacts of illness on children, they did not establish whether families affected by BD face unique challenges or if these common challenges manifest in distinct ways specific to the context of BD. Such generalizations could lead to misaligned intervention strategies. Given these shortcomings, there is a pressing need for a dedicated qualitative meta-synthesis to build understanding of parenting experiences specific to the context of parental BD. Such an approach can consolidate diverse clinical and cultural perspectives and offer a more rounded view of the challenges and needs of families.

Building upon Harries et al. (2023), who underscored the bidirectional nature of the parent–child dynamic in families with parental mental illness, our study aims to delve deeply into the parent–child relationships and interactions in the context of parental BD. This focus represents a more concentrated approach, honing in specifically on parents with BD, in contrast to earlier qualitative syntheses which broadly examined the experiences of parenting with serious mental illness (SMI) (Harries et al., 2023) or living with a parent with SMI (Källquist & Salzmann-Erikson, 2019). Acknowledging the limitations of relying exclusively on parent-only informants, as cautioned by Hagai Maoz et al. (2014) who highlighted the potential bias in parent-reported data on children’s psychopathology due to parental mood fluctuations, we include inputs from informants other than parents, including children, partners, and other relevant contributors. This strategy provides a holistic view of the familial impact of parental BD, and the insights gained will be invaluable for shaping policies, inspiring further academic discussion, and guiding clinical interventions.

## Methodology

Our review adheres to the Preferred Reporting Items for Systematic Reviews and Meta-Analyses (PRISMA) guidelines (Page et al., 2021). We submitted and received approval for our protocol from PROSPERO, registration number CRD42022322046, before commencing the review.

### Eligibility Criteria

We established specific criteria to select relevant studies:

1. Publication Type and Time Frame: We limited our selection to English-language, peer-reviewed articles. To ensure consistency in diagnostic criteria, we focused on studies published from 1994 onwards, aligning with the introduction of the DSM-IV.
2. Methodological Approach: We incorporated studies that primarily followed qualitative research designs or had qualitative components within a mixed-methods framework. Purely quantitative papers, reviews, and theoretical articles were excluded.
3. Study Demographics: Our emphasis was on research examining families with at least one parent diagnosed with BD. We were particularly interested in studies that shed light on the dynamics between parents and their offspring (under 19 years old), to understand the unique parenting challenges in this age group.
4. Special Considerations: We included studies that broadly covered parental mental illnesses but had clear segments where the experiences of parents with BD could be distinguished. We also included papers which collected data from families where children were already adults but offered specific historical accounts of parenting during the children’s younger years (under 19 years). When details about bipolar diagnosis or a child’s age were unclear, we actively sought clarification from the study authors. In cases where this information was not provided despite our inquiries, we made the decision to exclude those studies from our analysis to maintain clarity and specificity in our findings.

### Literature Search and Screening

We searched the following databases: Medline, Embase, PsycINFO (via Ovid), as well as SCOPUS and CINAHL, targeting articles published between 1994 and September 2, 2024. Our search dates were April 21, 2022, December 28, 2022, July 2, 2023, January 21, 2024, and September 2, 2024. We used a mix of key terms and their synonyms to explore the themes of "parents," "bipolar disorder," "children," and "qualitative research." Details of the search terms are available in Table 1. Further, we extended our search to bibliographies and citation indices of eligible articles via the Web of Science platform.

**Table 1:** The full version of the search terms (Medline^a^)

Concepts	Keywords
(1) Qualitative	((("semi-structured" or semistructured or unstructured or informal or "in-depth" or indepth or "face-to-face" or structured or guide) adj3 (discussion* or questionnaire*)) or (focus group* or qualitative or ethnograph* or fieldwork or "field work" or "key informant")).ti,ab. or interview*.tw. or interviews as topic/ or focus groups/ or narration/ or qualitative research/ or experience*.tw. or perception*.tw
(2) Bipolar	(BD or BD1 or BD2 or BDi or BDii or BDNOS or bi-polar or bipolar or manic or mania or hypomania or hypomanic or hypo-mania or hypo-manic).tw. or Bipolar Disorder/
(3) Parent	(parent* or mother* or maternal* or father* or paternal* or famil*).tw. or parents/
(4) Child	(child* or neonat* or newborn* or new-born* or infant* or baby or babies or toddler* or pre-school* or preschool* or school* or youth* or pre-adolescen* or adolescen* or teen* or kindergarten* or nurser*).tw. or adolescent/ or exp child/ or exp infant/
(5) = 1 and 2 and 3 and 4 (6) = Limit 5 to English (7) = limit 6 to yr = “1994–2023”	

^a^This example of search terms was used for searching one of the five databases (Medline) on April 21, 2022. For the search terms used in other databases or during different search phases, please contact the authors via email

Two team members, ET and HM, independently screened titles and abstracts, subsequently reviewing the full texts for relevance. The two raters demonstrated a strong level of agreement, evidenced by a Cohen’s kappa value of approximately 0.92. On the rare occasions where disagreements arose, they were effectively resolved by consulting additional team members, CC and KEAS. If articles had missing or unclear data, we contacted the original authors for clarification.

Our initial literature search was confined to English due to resource limitations for translation. Recognizing the potential of AI-assisted tools, we later extended our scope to non-English sources. Utilizing ChatGPT 4.0, the first author translated and included two additional non-English papers on January 21, 2024, thereby enriching our study’s cultural and linguistic diversity. To ensure accuracy, these translations underwent a rigorous back-and-forth translation procedure and were validated by a researcher fluent in Portuguese and Spanish.

### Data Extraction and Analysis

We imported excerpts of relevant content into NVivo software (version 1.71) for thematic synthesis, following the approach outlined by Thomas et al. (2008). Our analysis focused on the results sections of the selected articles to capture the lived experiences of parents and children in the context of parental BD. The coding process began with multiple thorough readings of the selected articles, allowing us to immerse ourselves in the content. We coded both direct quotations from parents or children and the authors’ interpretations that met our inclusion criteria. Codes were generated inductively, emerging directly from the text, with particular focus on those derived from direct quotations, as they offered immediate insights into the participants’ experiences.

After the initial coding, we grouped related codes into broader categories, which were extensively reviewed and discussed within the research team using the Miro platform (https://miro.com/). This collaborative process was crucial for refining the categories into overarching themes, ensuring they were not only grounded in the data but also reflective of the co-constructed understanding between the text and our interpretations. The final themes were guided by a critical realist epistemology, acknowledging the reality of social phenomena while recognizing the role of subjective interpretation. We adhered to the ENTREQ guidelines (Tong et al., 2012) throughout the analysis to ensure transparency and rigor.

### Quality Assessment

The quality of the studies included in this review was appraised using the 10-item Critical Appraisal Skills Program (CASP) Qualitative Studies Checklist (CASP, 2018). ET and HM independently conducted this evaluation. Where disagreements arose regarding the quality score, discussions were held to reach a consensus. It should be noted that the CASP tool itself doesn’t offer a cumulative score. For this reason, we employed a numerical scoring system, assigning values of 0 for ‘No’, 0.5 for ‘Partially Agree’, and 1 for ‘Yes’. This scoring system was adapted into categories of high (scores > 8–10), moderate (scores 6 to 8), and low (scores ≤ 5) methodological quality as delineated by (Harries et al., 2023). No eligible studies were removed from this meta-synthesis, as none of the studies displayed critical methodological flaws that would warrant their exclusion.

### Reflexivity Statement

Our research team, culturally and professionally diverse, is united in our focus on mental health and BD. The blend of our professional expertise and personal parenting experiences deeply informs our approach to the data. The lead author, ET, brings a multifaceted perspective to BD- as a psychiatrist, a family member of someone with BD, and a parent. This combination of professional and personal experience brought an understanding of the complexities of parenting in the context of BD. HM, transitioning from an experienced primary school teacher to a doctoral student, specializes in school-based mental health interventions. KEAS, a psychiatrist with expertise in mood disorders, and CC, an academic clinical psychologist focused on psychological therapies for youth, both served as supervisors to the lead author. The other team members, FL and SJ, are experts in developing family-oriented BD treatment programs. Our team includes four parents with children ranging from toddlers to young adults. This diverse range of parenting experiences allows us to bring the lived experiences of parenting under different circumstances to our analysis. The lead author was influenced by Eastern Asian culture whereas the other authors come from White/Western cultural backgrounds. We used reflective notes and in-depth team discussions to help ensure that all perspectives were balanced and included.

## Results

### Study Characteristics

**Fig. 1:**
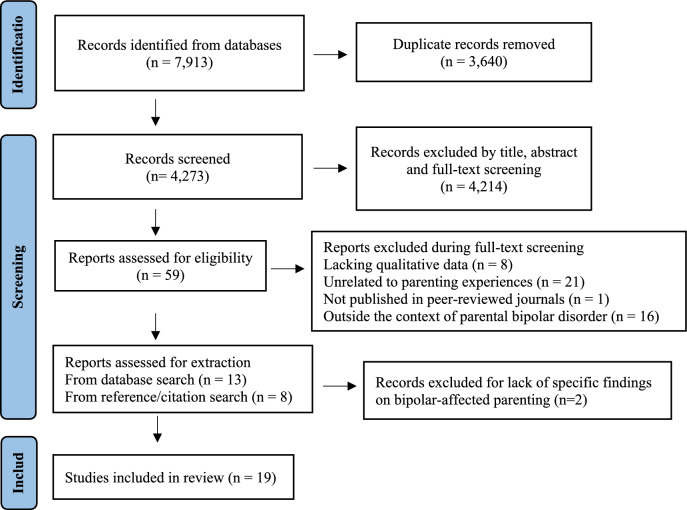
PRISMA Flow Diagram for the systematic review process

Study Characteristics: The systematic review included 19 studies, detailed in Table 2 and illustrated by the PRISMA flowchart in Fig. 1. This study includes perspectives on parenting experiences from various family roles, with five studies focusing on mothers, (Anke et al. (2019); Chen et al. (2021); Mulvey et al. (2022); Stallard et al. (2004); Venkataraman & Ackerson (2008)); one study centering on fathers, (Chen et al. (2023); three studies including both parents, (Bee et al. (2013); Tjoflat & Ramvi (2013); Wilson & Crowe (2009)); eight studies reporting on children’s perspectives, (Aldridge (2006); Backer et al. (2017); Campos et al. (2018); Liu et al. (2022); Petrowski & Stein (2016); Reupert et al. (2012); Venkataraman (2011); Vivanco & Grandon (2016), and two studies encompassing views from both parents and children, (Pattanayak & Sagar (2012) and Davison & Scott (2018). Across the included studies, a total of 54 mothers, 8 fathers, and 42 offspring met our criteria for informants. The included studies were geographically diverse, with 5 originating from North America, 2 from South America, 6 from Europe, 4 from Asia, and 2 from Australia and New Zealand. Regarding the assessment of BD diagnoses, the included studies employed various methods. Notably, none of the studies used a structured clinical interview to confirm diagnoses. Seven studies relied on documented diagnoses in clinical settings, while another five studies based their diagnoses on self-reports from participants. Seven did not specify their diagnostic methods. Our analysis generated 99 refined codes: 13 were from both parents and children, 52 were from parents (2 from fathers, 18 from mothers, 18 from both and 15 unspecified), and 33 from children. Table 3 summarizes the contributions of each included study to theme generation.

**Table 2:** The characteristics of studies included in the systematic review (*n* = 19)

Author, year, location	Study aim^a^	Sample description	Verification of diagnosis	Recruitment method	Data collection / analysis	Main themes	Eligible sample information
Anke et al. (2019), Norway	Investigate postpartum relapse risk perceptions among women with BD	Pregnant or postpartum women with BD I or II (*n* = 26)	Documented diagnosis	Mental health clinics, child services, well-baby clinics, and maternity wards	Semi-structured interviews / Inductive TA	- Perinatal concerns: Illness relapse; Early mothering; Perinatal impact on child; Illness impact on partner - Perinatal resources & preparations: Support network and personal strategies	Mothers with BD (*n* = 13), aged 25–37 (mean = 32.9)
Aldridge (2006), UK	Assess experiences of children caring for parents with mental illness	Parents with chronic mental illness, their caregiving children, and key workers	Not stated	UK’s young carers projects	“One-to-one interview” / Not stated	- Children’s care responsibilities: Role adaptation (not just role transference) - Positive outcomes: Caring help reinforce parent–child bonds - Family needs: Children’s care fills the gap in health and social care services - Parenting capacity: Mutable and self-perceived - Recognizing children’s contribution and needs	OPBD (*n* = 1)
Backer et al. (2017), UK	Examine how living with a parent with BD affects young children’s emotional health	OPBD (4–12 years old)	Self-reported	UK user-led BD organization	Computer-assisted, semi-structured interviews (In My Shoes) / TA	- Perception of parents: Parent with BD, ‘well’ parent - Knowledge and awareness of BD: Communication about illness, description of illness - Managing family life with a parent with BD: Emotional effects on child; ‘Independent’ child; Source of support; Avoidance and coping - Living in a family with BD	OPBD (*n* = 10), aged 4 to 10 years
Bee et al. (2013), UK	Assess quality of life for children in families coping with severe mental illness	Not stated	Self-reported	Advertisement or external emails	Focus groups (professionals) and open-ended interviews (children and parents) / Inductive TA	- Emotional wellbeing - Social wellbeing - Economic wellbeing - Family context and experiences - Self-esteem and self-actualization	OPBD (*n* = 1) and mother with BD (*n* = 1)
Campos et al. (2018), Brazil	Exploring adult perceptions of maternal BD diagnosed in their childhood and its implications	Adults whose mothers, now in psychiatric treatment, were diagnosed with BD during their childhood	Documented diagnosis	Adult Psychiatry outpatient clinic of the University Hospital	Semi-structured interviews / Content analysis	- Childhood: Caregiving burdens, guilt and insecurity - Adolescence: Maternal rejection, shame, social isolation, and premature adult roles - Adult life: Balancing personal sacrifices with coping strategies	Adult OPBD (*n* = 7)
Chen et al. (2021), China	Explore the parenting & family experiences of mothers managing mental illness	Mothers with current or past mental illness, with at least one child under 18	Self-reported	Social media advertising and online peer support groups	Semi-structured interviews / IPA	- Motherhood as a central identity - Stigma of Maternal Mental Illness - Impact of mental illness on parenting: Impact of fluctuating moods on parenting; Feeling guilty, overwhelmed, and helplessness; Self-acceptance - Perceptions about the impact of the mental illness on children: Hereditary worries; Impact on children’s development; Reducing the negative impact on children - Experiences of talking to children about mental illness - How having children impacts mothers’ mental illness and their recovery - Support obtained and needed: Helpful support; Unhelpful support; Support needed	Mothers with BD (*n* = 3, OPBD: 4–10 years)
Chen et al. (2023), China	Understand parenting & family experiences of fathers with mental illness	Fathers with current or past mental illness, with at least one child under 18	Self-reported	Social media advertising and online peer support groups	Semi-structured interviews / IPA	- Mental illness undermines fatherhood images - Parenting in the context of mental illness: Positive father-child interaction; Parenting disengagement; Harsh parenting; Inconsistent parenting - Concerns about the negative impact of their illness on children: Fear & Protection - Children as a burden and a source of hope - Stigma - Relying on family support: Isolation & connection; Further needs - Unmet professional and peer support needs: Lack of professional support; Professional/peer support needs	Fathers with BD (*n* = 3, OPBD: 5–12 years)
Davison & Scott (2018), USA	Explore attitudes toward interventions for OPBD	(1) Parents with BD I or II (*n* = 6, 2 fathers, 4 mothers, median = 48 years) (2) OPBD (*n* = 7, 3 boys, 4 girls, aged 12–26, median = 16, without psychological Issues)	Not stated	Parents with BD: A mental health conference or local clinical services; OPBD: Community professional network	Open-ended Interviews / TA	- Information and knowledge about BD - Parents with BD: Identification and modification of risk; Uncertainties and expectations for the future - OPBD: Concerns about Parents with BD; Acceptable models of support	As stated in the ‘Sample description’
Liu et al. (2022), Taiwan	Assess adult children’s perceptions of family resilience, its barriers, and cultural influences when a parent has BD	OPBD (*n* = 20, aged 20 or over, excluding those with parents having other mental illness)	Documented diagnosis	Acute psychiatric ward	Semi-structured interviews / IPA	- Family resilience: Ill parents try to be good parents; Parents’ personal strengths; Parents’ positive attitudes toward BD; Flexible family roles; Strong family bonds; families’ social connections - Barriers to family resilience: Poor parenting/family function, conflict between parents, poor mental health literacy	OPBD with quotes about experiences up to 18 years old (*n* = 4, comprising 3 females and 1 male)
Mulvey et al. (2022), USA	Explore motherhood experiences of criminally involved women	Mother with Axis I disorders and daily functional impairments on the SMI probation caseload (*n* = 48)	Documented diagnosis	Serious Mentally Ill probation caseload	Semi-structured life-course interviews / Unspecified inductive approach	- Normative mothering - Aspiring to break the cycle - Constrained mothering - Failure and state intervention - Children as parents - Children as a catalyst for change	Mothers with BD (*n* = 12), aged 20s-50s
Pattanayak & Sagar (2012), India	Understand the perspectives of patients with BD and their family members regarding family risk and genetic counseling	Patients with BD (*n* = 5); Family members of patients with BD (*n* = 11)	Documented diagnosis	Outpatient psychiatric clinic	Open-ended interviews / TA (Boyatzis, 1998)	- Emphasis on external/situational causes - Cultural beliefs govern causal explanation - Help-seeking: a shared decision - Perceived genetic risk low, yet worrisome - Concerns mainly about future generations - Desire to alter perceived genetic risk - Knowledge of precise risk both helpful and anxiety-inducing - Unmet need for preventive information	One mother with BD: (35 years); One father with BD (31 years)
Petrowski & Stein (2016), USA	Assess role reversal and obligation in young adult daughters; study family bonds and maternal mental illness impact	Daughters of a mother with long-term mental illness (*n* = 10, aged 18–22)	Daughter-reported	Online student research participation system and undergraduate courses	Semi-structured interviews / Content analysis	- Parentification - Felt obligation - Relationship with father and siblings - Positive growth	One 18-year-old daughter of a BD mother
Reupert et al. (2012), Australia	Explore the experiences of children with a parent who has a dual diagnosis	Children with a parent having dual diagnosis of mental illness and substance abuse (*n* = 12, aged 8–15)	Not stated	Through an organization focused on families with a parent having dual diagnosis	Semi-structured interviews / IPA	- Meaning of family - Understanding the parent and his or her illness - coping and reacting - preferred supports	One 8-year-old girl of a mother with BD and marijuana abuse; A 14-year-old boy of a father with BD, ADHD, marijuana & alcohol abuse
Stallard et al. (2004), UK	Assess parent and child perspectives on the impact of the parent’s mental illness	Parent with mental illness (*n* = 24, receiving outpatient care in CMHT) and their dependent children (*n* = 26)	Not stated	CMHT	Semi-structured interviews / Not stated	- Adult mental health team barriers: Time pressures and limited resources; Client-focused approach; Lack of child-/family-focused skills; Protecting the needs of the adults - Parental barriers: Adult needs dominate; Parental denial & fears; Protecting the children - Child barriers	Mother with BD (*n* = 1)
Tjoflat & Ramvi (2013), Norway	Experiences of parenting while managing BD	Parents with BD (*n* = 6; 5 mothers; aged 31–50; children under 18; No other mental illnesses)	Documented diagnosis	CMHT	Semi-structured interviews / IPA	- Balancing BD and parenting - Need for support versus perceiving stigma - Dependence on their children - Change and growth	As stated in the ‘Sample description’
Venkataraman & Ackerson (2008), USA	Assess parenting strengths, challenges, and needs in mothers with BD	Mothers with BD I or II (*n* = 10, 8 BD-I and 2 BD-II)	Documented diagnosis	CMHT and support groups	Semi-structured interviews / Constant comparison of the GT	- Strengths in parenting: Child importance; Positive traits; Lessons from own parents - Challenges in parenting: Impact of depression/mania; Swings in parenting; Discipline issues; Child-related stress - Service needs: Discipline help; Support groups; Crisis services; Managing child behavior; Other needs	As stated in the ‘Sample description’
Venkataraman (2011), USA	Explore the perspectives of children of mothers with BD in the parenting	OPBD without psychiatric diagnosis (*n* = 4, Aged 5–13)	Not stated	CMHT and support groups	Semi-structured interviews / Constant comparison of the GT	- Strengths in parenting: Helpful; Humor; Setting limits; Being available - Challenges in parenting: Oversleeping; Anger; Unpredictable moods; Dependency on children - Other finding: Fear of having or getting the disorder	As stated in the ‘Sample description’
Vivanco & Grandon (2016), Chile	Experiences raised by a parent with serious mental illness	Children (aged 18–29) of people with serious mental illness (5 schizophrenia, 6 BD)	Not stated	Referral by mental health facility staff	Semi-structured interviews and a group interview / TA	- Childhood: Fear, vulnerability; concerns for parent safety; uncertainty; sadness; and family protection - Adolescence: Family burden; guilt; role changes; identity formation; difficulty relating to parents; grief; feelings of abandonment and loss; impact of medication, preserving parental role; adapting to change - Young adulthood: Family loyalty; challenges in separating from the family; stigma	Adult OPBD (*n* = 5)
Wilson & Crowe (2009), New Zealand	How parents with BD view their parenting role	Parents with BD (*n* = 5, 4 mothers, 1 father)	Not stated	Not stated	Unspecified interviews, DSM-IV and popular texts / Critical discourse analysis	- Moderation - Monitoring emotional regulation	As stated in the ‘Sample description’

*ADHD* attention-deficit/hyperactivity disorder, *BD* bipolar disorder, *CMHT* community mental health team, *Dx* diagnosis, *GT* grounded theory, *IPA* interpretative phenomenological analysis, *MDD* major depressive disorder, *OPBD* offspring of parents with bipolar disorder, *SMI* severe mental illness, *TA* thematic analysis

^a^The focus here pertains to the original research aim of the study. This study focuses exclusively on extracting information related to the parenting experiences within families affected by bipolar disorder

**Table 3:** Matrix of theme representation within the included studies

Author (Year)	Codes	The Multifaceted Landscape of Parenting			The Evolving Dynamics of Child-Parent Relationships			The Dual Nature of Childcare for Parents with BD			Navigating Parental Challenges with BD		
		Parental Engagement	Parental Emotional Regulation	Parental Consistency	From Limited to Comprehensive Under-standing	From Reactive to Proactive Coping	From Compassionate to Overwhelmed Care	Childcare as Top Priority vs Self-Care	Childcare as Emotional Comfort vs Strain	Childcare as Motivation Promoter. Vs Underminer	Mindful and Proactive Strategies to Parent Children	Empathetic and Empowering Measures to Support Parent	Transparent and Tactful Communication to Strengthen Parent–Child Relationship
Aldridge (2006)	2		v						v				
Anke et al. (2019)	10	v		v				v	v				v
Backer et al. (2017)	28	v		v	v	v	v				v		v
Bee et al. (2013)	2					v	v						
Campos et al. (2018)	10		v		v		v						v
Chen et al. (2021)	29	v	v	v		v			v	v	v	v	v
Chen et al. (2023)	24		v	v			v		v	v	v	v	v
Davison & Scott (2018)	6				v				v		v		
Liu et al. (2022)	6					v	v		v				
Mulvey et al. (2022)	16	v	v	v	v	v	v	v	v	v			v
Pattanayak & Sagar (2011)	3										v		
Petrowski & Stein (2016)	5	v				v	v						
Reupert et al. (2012)	3		v			v	v						
Stallard et al. (2004)	3			v									
Tjoflat & Ramvj (2013)	24	v	v	v	v	v	v	v	v	v	v	v	v
Venkataraman & Ackerson (2008)	29	v	v	v		v	v	v	v	v		v	v
Venkataraman (2011)	19	v	v	v	v	v	v						
Vivanco & Grandon (2016)	8		v	v	v		v						
Wilson & Crowe (2009)	7							v	v			v	

### Quality Assessment

In our systematic appraisal of 19 selected studies, outlined in Table 4, we noted that the majority exhibited high (15 studies) or moderate (4 studies) methodological quality. Each study under review did not present any major methodological deficiencies that could impair the validity of their data for our qualitative synthesis. Hence, all studies were retained in our analysis. However, it is crucial to point out that only the studies by Bee et al. (2013) and Reupert et al. (2012) thoroughly considered the dynamics of power between researchers and participants. This is a critical aspect since unaddressed power imbalances and potential biases might influence participant responses and actions, generating concerns about the integrity of the research findings.

**Table 4:** Methodological quality assessment of included studies

First author (Year)	Clear statement of research aims	Appropriate use of qualitative methods	Research design aligned with research aims	Recruitment strategy suitable for research aims	Data collection addressing the research issue	Adequate consideration of researcher-participant relationship	Proper ethical considerations	Rigorous data analysis	Clear presentation of findings	Value of the research	Score
Anke et al. (2019)	Yes (1)	Yes (1)	Yes (1)	Yes (1)	Yes (1)	No (0)	Yes (1)	Yes (1)	Yes (1)	Yes (1)	High (9)
Aldridge (2006)	Yes (1)	Yes (1)	PA (0.5)	PA (0.5)	PA (0.5)	No (0)	PA (0.5)	PA (0.5)	PA (0.5)	PA (0.5)	Moderate (6)
Backer et al. (2017)	Yes (1)	Yes (1)	Yes (1)	Yes (1)	Yes (1)	No (0)	Yes (1)	Yes (1)	Yes (1)	Yes (1)	High (9)
Bee et al. (2013)	No (0)	Yes (1)	Yes (1)	Yes (1)	Yes (1)	Yes (1)	Yes (1)	Yes (1)	Yes (1)	Yes (1)	High (9)
Campos et al. (2018)	Yes (1)	Yes (1)	Yes (1)	Yes (1)	Yes (1)	No (0)	Yes (1)	Yes (1)	Yes (1)	Yes (1)	High (9)
Chen et al. (2021)	Yes (1)	Yes (1)	Yes (1)	Yes (1)	Yes (1)	PA (0.5)	Yes (1)	Yes (1)	Yes (1)	Yes (1)	High (9.5)
Chen et al. (2023)	Yes (1)	Yes (1)	Yes (1)	Yes (1)	Yes (1)	PA (0.5)	Yes (1)	Yes (1)	Yes (1)	Yes (1)	High (9.5)
Davison & Scott (2018)	Yes (1)	Yes (1)	Yes (1)	Yes (1)	PA (0.5)	PA (0.5)	Yes (1)	Yes (1)	Yes (1)	Yes (1)	High (9)
Liu et al. (2022)	Yes (1)	Yes (1)	Yes (1)	Yes (1)	Yes (1)	No (0)	Yes (1)	Yes (1)	Yes (1)	Yes (1)	High (9)
Mulvey et al. (2022)	Yes (1)	Yes (1)	Yes (1)	Yes (1)	Yes (1)	No (0)	PA (0.5)	Yes (1)	PA (0.5)	Yes (1)	Moderate (8)
Pattanayak & Sagar (2011)	Yes (1)	Yes (1)	Yes (1)	Yes (1)	Yes (1)	No (0)	PA (0.5)	Yes (1)	Yes (1)	Yes (1)	High (8.5)
Petrowski & Stein (2016)	Yes (1)	Yes (1)	Yes (1)	Yes (1)	Yes (1)	No (0)	PA (0.5)	Yes (1)	Yes (1)	Yes (1)	High (9)
Reupert et al. (2012)	Yes (1)	Yes (1)	Yes (1)	Yes (1)	Yes (1)	Yes (1)	Yes (1)	Yes (1)	Yes (1)	Yes (1)	High (10)
Stallard et al. (2004)	Yes (1)	Yes (1)	Yes (1)	Yes (1)	Yes (1)	No (0)	Yes (1)	PA (0.5)	Yes (1)	Yes (1)	High (8.5)
Tjoflat & Ramvi (2013)	Yes (1)	Yes (1)	Yes (1)	Yes (1)	Yes (1)	No (0)	Yes (1)	Yes (1)	Yes (1)	Yes (1)	High (9)
Venkataraman & Ackerson (2008)	Yes (1)	Yes (1)	Yes (1)	PA (0.5)	Yes (1)	No (0)	No (0)	Yes (1)	Yes (1)	Yes (1)	Moderate (6.5)
Venkataraman (2011)	Yes (1)	Yes (1)	Yes (1)	Yes (1)	Yes (1)	No (0)	Yes (1)	Yes (1)	Yes (1)	Yes (1)	High (9)
Vivanco & Grandon (2016)	Yes (1)	Yes (1)	Yes (1)	Yes (1)	Yes (1)	No (0)	PA (0.5)	Yes (1)	Yes (1)	Yes (1)	High (8.5)
Wilson & Crowe (2009)	Yes (1)	Yes (1)	Yes (1)	PA (0.5)	Yes (1)	No (0)	Yes (1)	PA (0.5)	PA (0.5)	Yes (1)	Moderate (7.5)

### Overview of the Results

**Fig. 2:**
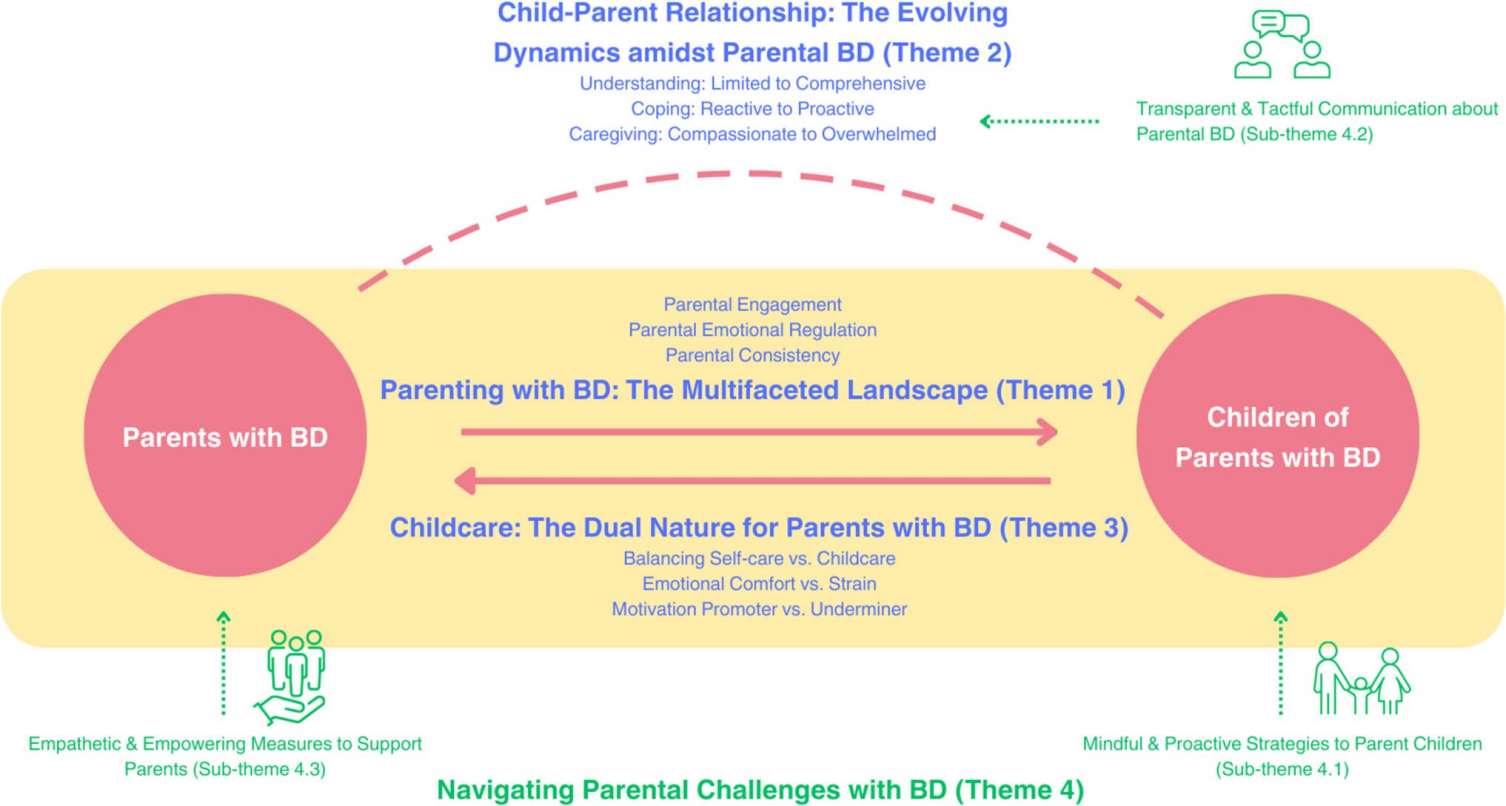
The conceptual model depicting themes and sub-themes

Our study highlights the nuances of parent–child interactions in the context of parental BD (Fig. 2). Theme 1 explores the multifaceted impacts of BD on parenting, influencing engagement, emotional regulation, and caregiving consistency. Theme 2 uncovers the evolution of child-parent relationships in the face of BD-affected parenting, showcasing children’s adaptive responses through progressive understanding, coping strategies, and caregiving roles across developmental stages. Theme 3 sheds light on the reciprocal effect of childcare on parents with BD, revealing its dual nature as both an emotional stressor and supporter, which paradoxically both distracts from and motivates self-care. Finally, Theme 4 identifies crucial support strategies for families navigating BD-related parenting challenges. This includes implementing mindful and proactive measures to lessen the effects of parental BD on children, fostering transparent and sensitive parent–child communication about BD to aid child adaptation, and providing empathetic and empowering support for parents with BD as they cope with parenting challenges.

### Theme 1: The Multifaceted Landscape of Parenting with Bipolar Disorder

The first theme investigates how BD affects parenting. It includes subthemes such as Parental Engagement and Emotional Regulation, which spotlight the immediate influence of BD’s acute mood states on parenting, moderated by factors like medication, positive traits, and mood phases like hypomania. Furthermore, Parental Consistency addresses the long-term challenges of maintaining consistent parenting in the face of recurrent mood swings, underscoring the vital role of a robust support system in mitigating the impact of parental inconsistency.

#### Subtheme 1.1: Parental Engagement

Participants articulated that major mood episodes associated with BD can markedly reduce parental engagement, disrupting familial stability and emotional connection. Fluctuating attention and energy levels contribute to this disengagement. A mother with BD described this experience: “*When you are depressed, you don’t have the energy. When you are hypomanic, you don’t have the time. So, it impacts you as a mother in both states*” (Anke et al., 2019, p. 5). Further detailing the impact, a parent elucidated "*I am so depressed, I am deprived of energy and I just sit there in the chair or sofa, and it is as if we are somehow not together… it is as if I am in my own world, pondering on things and then the children wonder why you are so distant.*" (Tjoflat & Ramvi, 2013, p. 82). Compounding factors like “*ruminations*” (Chen et al., 2021, p. 5) and emotional numbness also appear to play roles, with a mother’s description exemplifying the latter: "*I know there were days, times, periods that I was depressed enough so that `I just kind of went through life. I didn’t feel anything, I just, you know, did the grocery shopping, did the cooking, took care of their needs, but I wasn’t happy*" (Venkataraman & Ackerson, 2008, p. 397). Moreover, parental hospitalization for BD treatment can also contribute to temporary disengagement from children’s lives. This profound sense of separation and disconnection was described by a 6-year-old child whose mother with BD was hospitalized: "*It’s like being on the other side of the world with mommy*" (Backer et al., 2017, p. 218).

Children observed that parental disengagement associated with BD result in diminished emotional support: “*she [mother with BD] would still comfort, but not as much as maybe another mother would*” (Backer et al., 2017, p. 217). An 18-year-old reflected on the emotional gap: "*Everyone talks about, like ‘Oh, I can go tell my mom this.’ I can’t… I feel like it’s more like I’m the one missing out…*" (Petrowski & Stein, 2016, p. 2879). Others described how disruptions in children’s social and academic routines were directly linked to the impacts of parental BD. For instance, a 10-year-old daughter reported, “*I don’t really get to do much if she’s feeling down. But if she’s feeling happy, then I get to do lots of things when she takes me out.*” (Backer et al., 2017, p. 220). Moreover, parental BD impeded basic childcare and raised safety concerns, illustrated by a BD mother in a psychotic state: "*I started hearing voices and I was sick…my mind was going and my kids were with me…I didn’t know how to cook for them, I didn’t know how to do anything, so I just walked off*” (Mulvey et al., 2022, p. 1724).

Conversely, hypomanic phases can sometimes enhance parenting engagement, offering an increased energy that some parents find beneficial, evidenced by a mother’s reflection: "*having the hypomania helped because I am not sure I could have done all that if I hadn’t had that extra boost of energy*" (Venkataraman & Ackerson, 2008, p. 399), and another parent’s experience of shared joy during hypomania: "*we have a lot of fun together, we do things together that no other children experience*" (Tjoflat & Ramvi, 2013, p. 83). These reflections suggest that while BD presents significant challenges to parental engagement, there can also be moments where the elevated mood states associated with hypomania offer a temporary enhancement to the parental role.

#### Subtheme 1.2: Parental Emotional Regulation

Participants described mood disturbances in BD as deeply affecting parental behavior, particularly in managing irritability and impulsivity. A father described his mood as causing him to become “*easily irritated”* and “*wanting to physically hurt their child*” (Chen et al., 2023, p. 271). Similarly, a mother’s irritability was so acute that “*I was angry all the time at everything…everything she [the child] did just drove me crazy. Even if she sat on her dad’s lap, I was mad*” (Venkataraman & Ackerson, 2008, p. 397). Parental responses can escalate due to external stressors, exemplified by a teenager’s observation: “*when my mom gets stressed, she swears and hits the kids if they are naughty… we get yelled [at] a lot*” (Reupert et al., 2012, p. 157). Fatigue further exacerbates the issue, with a father noting that tiredness led him to “*negatively interpret the behaviors and emotions of his child*,” resulting in “*exaggerated reactions and intense anger*” (Chen et al., 2023, p. 271). During challenging bipolar episodes with psychotic symptoms, one mother experienced distressing misconceptions, where she “*inaccurately accused her son of molesting one of the younger children*” (Mulvey et al., 2022, p. 1724), while another admitted to “*transmit[ting] masculine energy*” through assaulting her son and harbored “*ideas of killing both herself and her son*” (Chen et al., 2021, p. 5).

Conversely, effective management of emotional symptoms through medication significantly enhances parental stability. One parent recounted the stabilizing effect of lithium despite some feelings of inhibition: “*My disease has leveled somewhat after I started with lithium, as it has made a tremendous difference; I don’t feel so depressed now because of the drugs that I take. I don’t feel either really happy or really bored. I may become sentimental, sometimes I cry, but it is not often, and it is not often that I laugh loudly and for long, either; it’s a bit inhibiting I think; yes, my kids should have experienced a little recognition, but on the other hand, I’m pretty balanced and I don’t feel angry. It would take a lot to make me angry, so there are both advantages and disadvantages* " (Tjoflat & Ramvi, 2013, p. 83). Medication has also been instrumental in reducing aggressive impulses, as noted by a mother whose inclination to act on such impulses diminished with treatment (Venkataraman & Ackerson, 2008, p. 397). However, the suppressive effects of medication are crucial to consider. A child reflecting on the complexity of medication’s impact on his mother with BD noted, “*I don’t know if it’s because of the illness or the medications, they’re bringing her sedated and she’s not the same person she was before, she was a happier person…with more energy*" (Vivanco & Grandon, 2016, p. 181).

Alongside medication, humor has been identified as a key emotional regulator for parents with BD; a 10-year-old’s observation highlights its efficacy reveals its role in mitigating parental mood swings: "*when she is angry or sad, sometimes something funny will happen, like something my brother says that will cheer her up (laughs)… she has a very good sense of humor*" (Venkataraman, 2011, p. 99). Furthermore, some mothers use humor to reframe their experience with BD, adding a layer of lightness to their emotional challenges. As one mother expressed, BD “has made life interesting” (Venkataraman & Ackerson, 2008, p. 395). These insights suggest that, in addition to medication, positive personality attributes like humor can be a valuable resource for parents managing BD.

#### Subtheme 1.3: Parental Consistency

Participants highlighted that parenting behaviors in BD can vary with the severity of mood fluctuations, creating a volatile environment for both the parent and child. Anke et al., (2019, p. 6) note that the extent of these impacts "*depends on the intensity of mood deviations*." The variability in parent–child dynamics is evident in a mother’s account: "*For one month I will just be awesome, and everything is done…and the next month just get away from me, leave me alone*" (Venkataraman & Ackerson, 2008, p. 400). This unpredictability permeates disciplinary approaches as well, with a group of mothers conceding to "*being inconsistent in enforcing rules with their children*," citing mood instability and cognitive impairments as primary causes (Venkataraman & Ackerson, 2008, p. 400). The unpredictable mood patterns associated with BD can significantly impact a parent’s ability to provide childcare. Stallard et al., (2004, p. 48) described a mother with BD “*At times she could not look after herself or the children*”. Additionally, Tjoflat & Ramvi (2013, p. 84) reported that several parents acknowledged their need for "*additional support in caring for their children*", underscoring the difficulties faced in managing parenting responsibilities amidst BD’s fluctuating moods. Moreover, the non-affected partner is not immune to these challenges; for example, a four-year-old observed his father to be “*angry and stressed*” while navigating familial responsibilities during the mother’s period of instability (Backer et al., 2017, p. 218).

In contrast, a robust support system is essential for maintaining parenting consistency among families with BD. It compensates for fluctuating parenting roles and is important in providing access to support, helping to prevent mood changes from escalating into severe episodes. Partners play a critical role of "*supervising their [parents with BD] parenting behaviors when ill*", particularly during emotional challenges, as a father with BD recounted (Chen et al., 2023, p. 273). Furthermore, partners often assume the primary caregiving role in the absence of the parent with BD. This dynamic is illustrated by the experience of a 7-year-old: "*Daddy’s normally with us, looking after us by himself, especially when she [mother with BD] went to the hospital*” (Backer et al., 2017, p. 218). The support extends further as evidenced by a young adult’s memories of extended family stepping in during a parent’s unwell periods: “*I remember when I was a little kid and Mom did not feel well, my grandfather would come and take care of us for a week or more. The brothers and sisters from both sides of my father and mother would help us*” (Liu et al., 2022, p. 181). Non-familial networks, or "*friend families*", further reinforce this system, with one mother expressing gratitude: “*you do have people that are not your blood family, but they are your friend family…we are fortunate*” (Anke et al., 2019, p. 8). Combining these insights, it is evident that while BD presents challenges to consistent parenting, the surrounding support from partners, family, friends, and community resources can provide the necessary structure and stability to navigate this fluctuation.

### Theme 2: The Evolving Dynamics of Child-Parent Relationships Amidst Parental Bipolar Disorder

In the context of parent BD, parent–child interactions evolve with the child’s age, developmental stage and exposure to the disorder. As time progresses, children’s understanding, adaptation, and caregiving roles related to their parent’s BD continuously transform and mature. Throughout different developmental stages, children may experience distinct emotional and practical challenges as they adjust to living with a parent managing BD.

#### Subtheme 2.1: From Limited to Comprehensive Understanding

Children’s grasp of BD typically evolves with age, with marked differences in understanding and engagement. Backer et al., (2017, p. 217) note that children aged 4–6 years often remain outside discussions of BD and exhibit no inclination to inquire about it, leading to situations where a six-year-old cannot understand why his mother "*just cries for no reason*", blame themselves for “*making his mother angry*”. From 7–10 years, children may have a growing curiosity about BD and a basic understanding, using terms like “*unwell*”, “*being ill*”, “*a mental illness called bipolar*,” and may even start to consider the possibility of developing BD themselves. For example, a 9-year-old boy reflected on this concern, saying “*He [father with BD] thought I had it… I was having more and more things to do with Bipolar and he’s told me that I might have it”* (Backer et al., 2017, p. 219). As children mature and their awareness of BD grows, they begin to confront more complex feelings. A 10-year-old described her anger when her routine was disrupted because of her mother’s BD: “*if we plan to go to a place and all of a sudden, my mom gets stomachache or irritable. I get quite angry cos we’ve already planned it and I’ve been looking forward to it*” (Backer et al., 2017, p. 220). Young adults reflecting on their childhood recalled anxiety about the possibility of a parent’s relapse: “*I was always very worried. When I noticed that she was a bit weird…I already noticed it. I was the first to notice, even as a child.*” (Campos et al., 2018, p. 41), and sadness due to changes in a parent’s personality from BD: “*there was a change in her. When she got sick…there was a change in terms of her personality, her way of reacting. It was very heartbreaking for me, I mean, it took me years to recover from that experience*” (Vivanco & Grandon, 2016, p. 179).

Adolescents’ developing understanding of parental BD, is often confounded by unpredictable mood fluctuations. One adolescent articulated their perplexity: "*You couldn’t tell if she was okay because she oscillated between love and anger*" (Campos et al., 2018, p. 42). Adolescence brings a complex mix of emotions along the journey of understanding parental BD, including shame and anger. A young adult recalled: “*I didn’t want anything to do with my mother…she was crazy, I was angry with her*” and added, " *Sometimes she [mother] didn’t take care of herself…didn’t do her hair, didn’t put on clothes, or wanted to wear inappropriate clothes … without understanding the problem I felt embarrassed*…" (Campos et al., 2018, p. 42). Concurrently, teens may gain further insight into how parental BD shapes their own identities. For example, a 15-year-old linked a unique aspect of her character to their parent’s BD: “*sometimes I act really different…I think I inherited a bit of her mood disorder*," embracing this positively: “*It makes me who I am and makes me a unique kind of person*” (Venkataraman, 2011, p. 103).

#### Subtheme 2.2: From Reactive to Proactive Coping

Children’s responses to parental emotional challenges also appear to be age dependent. Young children may act out with "*fighting and being naughty*" or feel “*scared when the parent was angry*” (Backer et al., 2017, p. 217). Emotional disengagement is another response to parental aggression; as one mother with BD observed, “*If I’m punching the wall, or I scream, … she’s [the child] just not there emotionally*” (Bee et al., 2013, p. 5). With maturity, coping methods advance; a 9-year-old learned to keep their distance: “*if she [mother with BD] is angry then I know to…avoid her slightly*” (Backer et al., 2017, p. 222). However, a 15-year-old described cautious engagement: “*I just kind of sit back and listen to how she … reacts to [others] and then if I have something I have to ask I ask if she is in a good mood… [If in a] bad mood I really don’t want to ask her because her first word is going to be, ‘No.’ I mean, even if it is a small thing like ‘Can I take the dog for a walk?’No’*” (Venkataraman, 2011, p. 101).

In addressing childcare challenges due to parental BD, younger children often turn to other family members for support. A 4-year-old reassured her mother with BD by saying: “*Mom, you can stay at home [to rest]. I’ll play with grandma*” (Chen et al., 2021, p. 5). As they grow, children increasingly manage self-care and assume caregiving roles. A 7-year-old demonstrated self-care by managing his routine amidst parental depression: “*I go downstairs, play, decide when I have my breakfast, make the breakfast… wait until daddy’s down*” (Backer et al., 2017, p. 221). An 8-year-old described providing sibling care: “*I help look after my little sister when mom needs a break*” (Reupert et al., 2012, p. 157). A teenager described overseeing home safety “*when her mother slept*” amid depressive spells (Venkataraman, 2011, p. 100). In some cases, these roles extend to crisis management. A mother with BD acknowledged her 10-year-old daughter’s role in “*ensuring that [her mother] was taking her psychotropic medication every day, policing any mental health crisis [mother] may have, and also trying to protect her during it”* (Mulvey et al., 2022, p. 1729)*.* Teenagers may also confront familial challenges. One described intervening against her violent father with BD: “*My father was also violent to my mom; that made me feel very angry…I urged my mother to leave him… I frequently argued with him*” (Liu et al., 2022, p. 180). Furthermore, an 18-year-old described defending her mother from social stigma: “*I feel like I have to defend her [mother with BD] when people talk about her*” (Petrowski & Stein, 2016, p. 2877).

Besides age, the duration of exposure to parental BD significantly influences children’s adaptation. A girl remarked, “*I’ve probably got used to it [parental BD] when I turned ten*” (Backer et al., 2017, p. 219). A 13-year-old boy mused about his mother’s long-standing BD, stating: “*She [mother with BD] has probably had it longer than you have been alive, and so you have probably actually grown up with it, except you haven’t known it, and so, can I go make a big deal about nothing?*” (Venkataraman, 2011, p. 103).

#### Subtheme 2.3: From Compassionate to Overwhelmed Care

Children’s instinctive readiness to assist their parents can manifest as joy, exemplified by a 10-year-old: “*When my mom feels irritable, but I know it’s not her fault…I feel happy because I like helping her [mother with BD]*” (Backer et al., 2017, p. 221), a quest for significance, as expressed by a 15-year-old: “*I would like to have responsibilities because I feel like needed*” (Venkataraman, 2011, p. 101). However, in the absence of a robust support network, parental reliance on children may intensify, as a widowed mother acknowledged: “*since my husband has passed away, I make a lot of excuses for her. Well, she (daughter) has got to be right next to me all the time*” (Venkataraman & Ackerson, 2008, p. 394). This often results in a role reversal, as a young adult recalled from her adolescence: “*So, the roles changed, and this marked me a lot, because the roles, indeed, changed, I became the mother and she practically my daughter. I was… the mother in my house. In every way, I took care of the house chores, the shopping, the money, how the house was managed…I bathed my mom, combed her hair, everything…*” (Vivanco & Grandon, 2016, p. 181).

Parents with BD are aware of the emotional burden their children bear in caregiving roles, as one parent with BD reflected: “*They [children] are supposed to look after for me, protect me, and that’s not good*” (Tjoflat & Ramvi, 2013, p. 86). These roles can cause varying levels of emotional distress in children. A teenager expressed feelings of annoyance: “*I will be wanting to go outside, she will go ‘Can you please just wait for me to take the shower?’… I just feel like, ‘Why do I have to be here… for you to take a shower… Come on mom, why don’t you take one on your own.*’” (Venkataraman, 2011, p. 102). The anxiety over a parent’s well-being was echoed by another child, as reported by a mother: “*she [the child] is like ‘Get up!’ You haven’t taken your pill yet…you’re gonna act up, and you’re gonna leave us*” (Mulvey et al., 2022, p. 1729). This caregiving strain can lead to depression, as a 15-year-old described after her mother’s suicide attempt: “*It just… kind of affected me slightly mentally, having to deal with what she’s like. Like, past like attempts of her trying to take too many pills, like and sort of how to keep her calm. It’s hard. The doctor’s said I’m depressed*” (Bee et al., 2013, p. 4). An adolescent recounted the turmoil of managing a turbulent family life: “*I had to take care of my mom and also worry about my father’s [with BD] drinking problems and debts…I had run away from home several times*” (Liu et al., 2022, p. 182).

### Theme 3: The Dual Nature of Childcare for Parents with BD: Balancing Challenges and Rewards

BD introduces a complex dynamic to parenting, influencing both parent–child interactions and the internal experiences of the parents. As these parents navigate the intricate balance of personal energy distribution, emotional responses, and motivations, a duality emerges, casting shadows and light on the complex journey of parenting with BD.

#### Theme 3.1 Childcare as Top Priority vs Self-Care

Parents with BD must constantly balance self-care with parenting demands. Limited time and energy often intensify this balancing act. One parent captured the tension, noting, “*if I am not in a good space, then I have to use all my energy just to look after me and cope*” (Wilson & Crowe, 2009, p. 880). Another parent underscored the essential nature of self-care, stating, “*I think there needs to be, like, a place where we could take our kids, to take them somewhere, because we need time to ourselves, but I mean for just bipolar*” (Venkataraman & Ackerson, 2008, p. 404). Despite personal challenges, many parents with BD described prioritizing their children, often valuing time with their children over taking breaks: “*I value most my time with them [children]. I love being with them. I don’t ever want vacations from them*” (Mulvey et al., 2022, p. 1722). This commitment is evident in expressions like “*my children are the most important*” and “*I push myself as much as I can… it hurts not to have enough strength for my children*” (Tjoflat & Ramvi, 2013, p. 82). Highlighting energy management, a mother with BD noted, "*There are things that have to be done, but it’s important that I don’t use all my energy dealing with them. The most important, is to use my time with my child, and with us as a family. I need to have the energy for that*" (Anke et al., 2019, p. 9).

#### Theme 3.2 Childcare as Emotional Comfort vs Strain

The parent–child bond often serves as an emotional anchor for parents with BD. Their children have been described as "*little suns who are energetic and happy*" (Chen et al., 2021, p. 6), symbolizing “*positive emotions*” and bridges of “*connection*” (Chen et al., 2023, p. 272). Support from children is evident in comments like, “*I make her [mother with BD] laugh…She’ll forget all about it, and then I know I’ve helped*” (Aldridge, 2006, p. 82) and “*when my father was in the hospital, I would share interesting things that happened at school*” (Liu et al., 2022, p. 81). Routine childcare soothes parents’ minds, as one noted: “*it [childcare routines] keeps my symptoms down too because I feel important. I feel like I’m needed*” (Mulvey et al., 2022, p. 1731). Such feelings are magnified during parenting successes, with a parent sharing, “*My children were doing so well… this is actually a victory for me, I am not worse than other persons, even though I am ill*” (Tjoflat & Ramvi, 2013, p. 86).

However, the challenges of BD can amplify guilt in parents over their irritability and need for solitude: “*I feel guilty a lot of the time because I get irritable with them*” (Wilson & Crowe, 2009, p. 880) and “*I just need to be alone, and that weighs heavily on my conscience, because I have chosen to have a husband and a child*” (Anke et al., 2019, p. 6). This is exacerbated by self-doubt, with a parent admitting: “*I felt really useless as a parent*” (Wilson & Crowe, 2009, p. 880). The strain intensifies when parents with BD see their child’s emotional distress: “*he [the son] said ‘you and your bipolar… you’ve ruined my life’… I couldn’t sleep that night…, I felt so worried and guilty…cos I was responsible…*” (Davison & Scott, 2018, p. 1115). When both parent and child face emotional turbulence, the relationship dynamics complicate, as voiced by a parent with BD, “*the friction between us is harder… it just does not mix*” (Tjoflat & Ramvi, 2013, p. 402).

#### Theme 3.3 Childcare as Motivation Promoter vs Underminer

Active participation in their children’s lives can give parents with BD a sense of purpose. Tjoflat & Ramvi (2013, p. 85) quoted parents with BD who had increased motivation in the context of enduring depressive symptoms: “*I know that I have kids to wake up for every day…instead of just sitting down and going to sleep, I wait for them to come home from school*.”, and to help cope with self-harm and suicidal tendencies: “*without my children, I would not be here, it’s that simple; thinking about them saved my life.*” Furthermore, aiming for a brighter future for their children, a father with BD was motivated toward “*treatment and self-growth”*, as he *“hoped to provide them with a good life experience*” (Chen et al., 2023, p. 272). However, separation from children can elicit varied responses from parents, either having increased motivation to pursue treatment to “*bring back my child*” from other family members (Chen et al., 2021, p. 6) or triggering despair around finding purpose, as conveyed by a mother with BD who admitted, “*I just felt like I’d rather die than not be able to, and to lose them all over again*” (Mulvey et al., 2022, p. 1727). In summary, children’s presence often gives parents with BD purpose and strength, while their absence can heighten emotional distress, prompting healing efforts or deep sorrow.

### Theme 4: Navigating Parental Challenges with BD

The previous themes highlight the impact of parental BD on families, while this theme emphasizes their critical needs for support. Parents with BD seek understanding and empathy, but stigma and judgment often hinder access to the help they need. Children require tailored support to navigate the risks and uncertainties, including potential hereditary effects. While communication about BD can help children understand and prepare for their parent’s BD, parents may face challenges in engaging in these conversations due to the disorder.

#### Subtheme 4.1 Mindful and Proactive Strategies to Parent Children

Parents with BD described mindful strategies to better understand and navigate the disorder’s impact on their children. For instance, a parent with BD shared a process of self-reflection, “*I spend a lot of time analyzing myself, trying to work out whether…I am just doing things on… an emotional level or… a more sort of logical level*” (Wilson & Crowe, 2009, p. 881). Another father with BD valued his child’s reactions as a gage for his parenting effectiveness, stating: “*He [child] is a good mirror. Because I care so much about him that I put more effort into reviewing all my parenting and his reactions*” (Chen et al., 2023, p. 272). These parents actively seek support to ensure their children’s well-being. Stallard et al., (2004, p. 48) describe a situation where a mother with BD, concerned about her own anger, feared for her children’s safety. In her words, “*when she [the mother] became angry, she expressed a fear that she might seriously harm her children. She felt her children were at risk and had asked social services to look after them [the children]*”. A mother with BD expressed the need for professional guidance on methods to manage the effects of her mood problems on children, “*Let’s say I get angry at my child … how can I manage that with my child? Or is it okay if I don’t deal with it? If I need to deal with it, then what should I do? … What do parents in our situation need to be aware of when we are with our children?”* (Chen et al., 2021, p. 7).

Parents with BD, aware of the disorder’s hereditary risks, show heightened concern about its transmission to their children, as one mother expressed, “*so afraid my child had inherited my illness*” (Chen et al., 2021, p. 5). These parents become particularly attentive to early signs of BD in their children and strive to differentiate early BD symptoms from normal behaviors. Davison & Scott (2018, p. 1114) described a parent with BD who conveyed, “*I spend too much time trying to work out if anything he does is a sign or a symptom… it stresses me out… I just wish someone would tell me when I can ignore it and when I need to start to worry*.” Moreover, some parents emphasize the value of early diagnostic methods, like genetic testing, yet often seek a level of certainty that may not currently exist. One parent articulated, *“…if I knew which of my kids was at risk then I could do something surely to stop it happening… maybe we could move house or change our lifestyle or do something to reduce the chances that it (onset of BD) would happen…*” Additionally, there is a strong emphasis on fostering a nurturing environment to possibly mitigate these risks, even as they navigate their uncertainty about how to best protect their children. This sentiment is encapsulated by a father with BD, “*I am very sensitive to any complaint from my five-year-old son, especially after struggling with illness for so many years, now I do not want it to surface in my son…. I try to raise him in right manner, teach him healthy habits… if there is something else I can do, tell me….*” (Pattanayak & Sagar, 2012, p. 466).

#### Subtheme 4.2: Transparent and Tactful Communication to Strengthen Parent–Child Relationships

Children’s strong motivation to understand parental behaviors linked to BD is clearly demonstrated in instances like this child’s earnest inquiry to a parent with BD: “*why don’t you answer when we’re speaking to you, why don’t you hear*” (Tjoflat & Ramvi, 2013, p. 84). Participants described how open dialogue about parental BD can be supportive for children. By explaining that specific behaviors are linked to the disorder, a mother with BD revealed that children might "*understand that it’s not their fault that mom or dad has an emotional problem… [and] don’t blame themselves*" (Chen et al., 2021, p. 6). Such communication instills hope, as illustrated when the same mother told her daughter: "*Mom is on medication now, and then I’ll slowly calm down*" (Chen et al., 2021, p. 6). This also readies children for possible relapses, as highlighted by Backer et al., (2017, p. 219), "*their parents discussing their symptoms… knowing their parent’s mood so they could be prepared.*"

However, the intricacies of parent–child communication are further complicated by the mood symptoms characteristic of BD. As highlighted by Tjoflat & Ramvi (2013, p. 84), a parent admitted, “*my kids wonder and they want to talk, and basically it goes well, but sometimes it is so difficult, I have enough to deal with in my own head, I have too many thoughts, I don’t want to talk whenever they want*”. The same study revealed that parents with BD are aware of the potential for *“adding an additional burden on their children*” by openly discussing their condition. Additionally, some parents struggled to translate intricate psychiatric jargon into language children can grasp, leading them to question “*how to talk about it*” (Chen et al., 2021, p. 6; 2023, p. 272). This hesitation is further emphasized among fathers with BD, as highlighted by Chen et al., (2023, p. 271), who often see themselves as those who "*protect the family*" and serve as mentors to guide their children to “*explore the world*." This paternal self-image can complicate their openness about their condition, as evidenced by a father’s worry that disclosing his BD could undermine his authoritative image, leading to fears that “*it might make the mountain-like father figure collapse into a pile of crumbs*”.

#### Subtheme 4.3 Empathetic and Empowering Measures to Support Parents

Support for parents with BD in parenting is crucial, as one parent with BD emphasized, “*We should have had more help*” (Tjoflat & Ramvi, 2013, p. 84). Such support is often hindered by misunderstanding and stigma, leading parents to conceal their condition, thus limiting their access to vital support. Chen et al., (2021, p. 7; 2023, p. 273) revealed that parents with BD, regardless of gender, perceive their illness as either “*unacknowledged or unaccepted*” by partners and family. This perception can lead to a sense of isolation and hesitancy in seeking familial support. In professional contexts, parents with BD may also encounter judgmental attitudes. For instance, a parent with BD expressed feeling judged by a mental health professional during an appointment for their child: “*I felt like he [the professional] was blaming me – I was sitting there with this child that I had deliberately ruined*” (Wilson & Crowe, 2009, p. 881). The fear of judgment may prompt these parents to mask their symptoms to conform to societal expectations. As a parent with BD explained, *"you try to be a good parent…So you don’t want other people to know that you are not quite right at the time, so you try to hide it, pretend there is nothing wrong, sweeping it under the carpet."*(Wilson & Crowe, 2009, p. 881). Similarly, concerns about their children’s welfare can lead to further self-censorship, as a mother with BD confessed to withholding “*all my information”* because *“I don’t know if I’ll be the cause for my daughter being teased or bullied at school*” (Chen et al., 2021, p. 5).

The importance of empathetic understanding in support structures is crucial for these parents. A mother with BD highlighted the value of peer support groups, specifically those comprising individuals with shared experiences: “*I would have to say it has to be with others that have bipolar because you don’t want to get someone without bipolar who doesn’t understand the conflicts or stresses that a bipolar person goes through*” (Venkataraman & Ackerson, 2008, p. 403). Parents with BD have highlighted a need for self-compassion and empowerment. Reflecting on her internal struggles, a mother shared her journey toward self-compassion, revealing, “*I was possibly asking myself to be more perfect [as a mother]. I couldn’t accept that I wasn’t able to be extra good to her [the child] with all my heart and soul. In moments of such self-doubt, I’d rather not be with her…However, recognizing this harsh self-critique, I have been striving to find a more balanced perspective*.” (Chen et al., 2021, p. 5). Through the guidance of counseling, this internal dialogue culminated in a realization that "*it’s okay to be an 80-point mom when I’m not well*" (Chen et al., 2021, p. 6). On the theme of empowerment, a father with BD captured the essence of his needs, asserting, “*The first thing is [letting you know] ‘you’re normal’ and the second is ‘you can do it’*,” as noted by Chen et al., (2023, p. 274). Another mother articulated her pursuit of empowerment in her parenting journey, stating, “*I would just like to know how to be a stronger parent, ensuring my kids respect me. While honesty and openness with them are paramount, sometimes I need discernment on when to hold back*” (Venkataraman & Ackerson, 2008, p. 401).

Addressing the gender-specific challenges in supporting parents with BD is essential. Fathers with BD often face unique barriers in seeking support, possibly influenced by paternal role expectations and relational issues. For instance, one father avoided seeking familial support due to his “*strong personality*” and reluctance to “*worry my family*” (Chen et al., 2023, p. 273). This can result in isolation, with some fathers relying on their children as their link to the external world, as one father noted: “*when I wasn’t well… I need a connection from the outside world more than anything else. At that time my child was the one that I thought was more stable and secure in the world… for me*” (Chen et al., 2023, p. 272). In contrast, mothers with BD tended to seek support from family and friends, finding reassurance and practical assistance in their networks, as illustrated by a mother’s experience: “*my parents are close all the way. They live nearby, and it’s both for the sake of me and our child, and not the least for the sake of my husband. Because when I get ill, he goes to them and they make plans together, and I don’t have to think about it*” (Anke et al., 2019, p. 8).

## Discussion

This study is the first to specifically explore parent–child experiences in the context of BD using a qualitative synthesis approach. It intricately dissects the dynamics of families grappling with BD, with experiences reflecting four central themes. The first theme examines the multifaceted challenges of parenting in the context of BD, showing how mood swings associated with BD can affect parental engagement, emotional regulation, and consistency. This theme also encompasses compensatory mechanisms, such as effective treatment, coping strategies, and support networks in mitigating the impacts of BD. Moving to the second theme, we chart the progressive deepening of children’s understanding of BD from basic awareness to more complex self-reflection, including their emotional journey and the shift from passive to active coping strategies when living with a parent with BD, as well as experiences of both empowerment and emotional strain due to early caregiving roles. Theme three contrasts the potentially competing demands of parenting and self-care among parents with BD. It highlights that parent–child relationships, while being a stabilizing and motivating force, can also lead to self-criticism during challenging periods. The fourth theme shifts focus to targeted strategies aimed at alleviating the effects of BD on key domains- self-regulatory and proactive strategies for child well-being, open and affirming communication for the parent–child bond, and empathetic and empowering support for the parents themselves. Overall, this research advances our understanding of how BD affects parent–child interactions. It underscores the dynamic evolution of these relationships across various stages of BD and child development, highlighting the need for tailored support that addresses the unique needs of both parents and children navigating the complexities of parental BD.

Our research explores the distinctive parenting challenges in BD. Echoing findings from qualitative syntheses on parenting across serious mental illnesses (Dolman et al., 2013; Harries et al., 2023), we observed the tension between parental self-care and childcare, along with the dual emotional and motivational effects of childcare, suggesting that these issues are common across various major mood and psychotic disorders. This underscores the importance of incorporating childcare considerations into support strategies for families affected by BD. While parental withdrawal and emotional regulation difficulties were also noted in Harries et al. (2023), our study indicates that parental inconsistency may be a particularly pertinent feature of BD. Furthermore, while Harries et al. (2023) described child emotional distress and parent–child role reversals in response to parental severe mental illnesses, our study provides more in-depth insights into parent–child interactions in the context of parental BD, including how children understand and cope with, and even support, parental BD as they age.

This study highlights the significant challenge of consistent parenting in parents with BD, driven by fluctuating behaviors and capacities due to mood swings. Children of these parents may experience ongoing inconsistency because BD involves two levels of mood fluctuations: (1) Episodic shifts between euthymic, subsyndromal, or full-blown manic and depressive episodes (Joffe et al., 2004), and (2) moment-to-moment and day-to-day mood lability, involving both negative and positive emotions, even outside bipolar episodes (Mildiner Moraga, 2024; Sperry & Thomas, 2022). This mood instability and variability across different timescales is more characteristic of BD than unipolar depression, such as MDD (Angst et al., 2003; Bartoli et al., 2024). In addition, fluctuations in functional capacity in BD (Proudfoot et al., 2014) contrast sharply with the more chronic course seen in schizophrenia (Flaaten et al., 2023). Our study highlighted how children can respond with anxiety and confusion to parenting inconsistency. Negative impacts on children’s emotions and behaviors are echoed in general population studies. For example, longitudinal research has shown that parental inconsistency can predict depression, aggression, and behavioral issues in adolescents (Carrasco et al., 2015; Surjadi et al., 2013). Further research is needed to explore the effects of parenting inconsistencies on children and families and mixed-methods studies will be crucial for improving our understanding of how mood variability in BD across different time scales impacts parenting and parent–child interactions. These insights could guide the development of personalized support programs and improve systems to better meet the dynamic needs of BD-affected families.

Another key finding is the evolving nature of parent–child interactions as children grow older, particularly in how they understand, react to, and cope with parental BD. The more comprehensive understanding and proactive coping strategies observed in older children and adolescents may be linked to several factors: prolonged exposure to parental BD, improved cognitive functions such as emotional recognition and regulation (Khawar et al., 2014; Sanchis-Sanchis et al., 2020), and a growing desire for autonomy during adolescence (Sebastian et al., 2008). However, adolescents are at higher risk for depression, more severe anxiety, and greater impairment in school functioning than younger children (Baker et al., 2021; Waite & Creswell, 2014). In addition, older offspring are more heavily impacted by caregiving roles due to increasing demands to provide emotional support for their family, alongside growing school and social responsibilities (Jurkovic, 1998) and the ongoing process of separation-individuation (Kins et al., 2012; Shah et al., 2023). In keeping with these reflections, the wider psychological intervention literature suggests that interventions for children and adolescents must be developmentally appropriate, considering factors such as age-specific language, tasks, and treatment delivery (Baker et al., 2021; Waite & Creswell, 2014). Our work builds on these insights, highlighting the need for age-specific interventions tailored to children’s developing comprehension, emotional responses, coping strategies, and caregiving roles in families with a parent with BD. For younger children, interventions might center on helping parents decode the emotions behind, and respond effectively to, children’s behavioral issues, alleviating child guilt stemming from misconceptions about parental mood fluctuations through age-appropriate communication, and ensuring that support systems are effective when parents are affected by BD. For older children and adolescents, strategies might include fostering adjustment to parental emotional states, addressing emotional reactions to BD’s impacts, identifying early signs of youth mental health problems, and mitigating excessive caregiving responsibilities.

Our findings suggest that families with a parent who has BD may benefit from multifaceted support, including parental mood management, support around parent–child interactions and parenting practices, and with specific communication around BD. This builds on previous findings, including intervention studies which have demonstrated the feasibility and positive impacts of addressing some of these areas. First, previous studies has highlighted that BD disrupts family functioning and increases caregiver burden (Reinares et al., 2016), and broader research shows reciprocal effects between parent and child psychopathology (Gunlicks & Weissman, 2008; Wesseldijk et al., 2018), underscoring the potential value of addressing parental BD through direct treatment, caregiver support (Reinares et al., 2008), and peer networks (Kelly et al., 2019). Second, the IBPI program, which focuses on parental well-being and positive parenting through a parent-only approach (Jones et al., 2017), and the RUSH program, which addresses parenting stress and parent–child dynamics via separate group sessions for parents and children (Resendes et al., 2023; Serravalle et al., 2021), have shown positive mental health benefits in at-risk children. For at-risk adolescents with non-BD mood disorders, family-focused therapy, promoting family communication skills and problem solving, has led to longer remission periods and reduced suicidal ideation among offspring (Miklowitz et al., 2020a; Miklowitz et al., 2020b; Weintraub et al., 2022). Third, Beardslee’s Family Talk Intervention, which facilitates parent–child conversations about parental depression, has demonstrated sustained improvements in children’s understanding of the illness, along with reductions in child anxiety, emotional symptoms, and improvements in prosocial behaviors (Beardslee et al., 2003; Bearslee et al., 2007; Solantaus et al., 2010). A similar approach may benefit families affected by parental BD. Overall, existing evidence suggests these multifaceted approaches could be advantageous for families with a parent with BD. However, comprehensive studies are needed to determine which strategies, structure, and timing are most effective across diverse family contexts. Such research would allow for tailored interventions, adapting support to each family’s unique needs.

Our study reveals significant gender-based differences in the experiences of parents with BD, with a particular focus on the unique challenges faced by fathers. The limited representation of fathers with BD in our study highlights a critical research gap. One of the included studies, Chen et al. (2023) conducted in China, highlights how paternal and masculine images may deter fathers with BD from discussing their condition openly with their children, driven by a fear of undermining their paternal image- a concern not notably expressed by mothers in our study. Other common barriers to BD disclosure, such as uncertainty about how to communicate the condition, worry about burdening children, and being impacted by mood symptoms, are shared challenges for both mothers and fathers. This is echoed by the qualitative findings of Reupert & Darryl (2009), conducted in Australia, which also underscored the importance of the self-image of "breadwinner" and "significant adult" to fathers’ identities. In contrast, mothers with BD tended to assume the primary caregiver role and were more likely to seek support from family and friends, as they navigated their illness. Given these findings, it is evident that more research focusing on the parenting experiences of fathers with BD is needed. Such studies would help determine whether fathers have distinct support needs based on their specific parental roles, particularly considering the cultural context of the existing research.

This study’s limitations merit consideration. The heterogeneity in study methodologies may shape theme generation. Additionally, the synthesis process may have resulted in the loss of nuanced context, particularly due to the overrepresentation of Western perspectives and the underrepresentation of fathers’ experiences. Notably, cultural differences in managing parental BD crises appear to be significant. In Western contexts, there is commonly a reliance on social services to safeguard the family unit, but this may amplify anxieties about potential family separation (Stallard et al. (2004); Mulvey et al. (2022); Harries et al. (2023)). In contrast, East Asian studies highlight family involvement during episodes, reflecting Confucian values prioritizing family obligations (Chen et al., (2021, 2023); Liu et al. (2022); Zhao et al. (2017). These variances underscore diverse recovery processes and support mechanisms in BD families across cultures. Our research team’s particular cultural perspectives may have influenced the data interpretation. For example, the lead author’s East Asian values emphasize support from extended family, potentially shaping the analysis toward these norms over others. Additionally, we may have overlooked cultural nuances from other backgrounds, such as those relevant to Black participants highlighted in Mulvey et al. (2022). As mental health clinicians and parents, our focus on supporting parents with BD might have overshadowed other aspects which may have been prioritized by others (for example, child protection professionals may have brought a different perspective). To mitigate these influences, we held regular team discussions to ensure a more balanced and comprehensive interpretation, considering a broader range of cultural contexts.

Owing to insufficient data on gender-specific parenting, our analysis could not investigate the distinct parenting approaches toward sons versus daughters among parents with BD, nor the parent–child interactions with boys and girls, despite literature suggesting that such differences could significantly influence child development outcomes (Morawska, 2020). Additionally, the methods used to assess BD diagnoses in the included studies varied, with some relying on self-reports and others not specifying their diagnostic methods. The lack of structured diagnostic approaches and dimensional measures may limit the strength of the findings. Including participants who are not accurately diagnosed with BD could introduce experiences that do not genuinely reflect those of individuals with BD. This could affect the credibility of the study and complicate the interpretation of the data, potentially weakening the overall robustness of the conclusions drawn. Lastly, the cross-sectional design of included studies captures only a momentary view of highly variable emotional states and parenting experiences in BD, which may fluctuate over time. Moreover, interviews with young adults recalling their childhood and adolescent experiences may include interpretations influenced by hindsight. Future research should, therefore, include longitudinal perspectives from both parents and children to more accurately reflect these dynamic experiences. Despite these limitations, the study’s strengths include strict selection criteria, a multidisciplinary research team, and the incorporation of comprehensive parent–child perspectives, which contribute to the depth and breadth of our understanding in this domain.

## Conclusion

Our review highlights the complex challenges experienced by parents with BD and their children. The fluctuating parenting behaviors and the children’s evolving responses necessitate targeted, flexible, and age-appropriate interventions. To develop a comprehensive and globally relevant support framework for families with a parent with BD, future research should further explore the cultural and gender-specific nuances influencing the effects of BD on parenting.
